# Nogo-A couples with Apg-1 through interaction and co-ordinate expression under hypoxic and oxidative stress

**DOI:** 10.1042/BJ20130579

**Published:** 2013-09-27

**Authors:** Florian Kern, Ruslan I. Stanika, Bettina Sarg, Martin Offterdinger, Daniel Hess, Gerald J. Obermair, Herbert Lindner, Christine E. Bandtlow, Ludger Hengst, Rüdiger Schweigreiter

**Affiliations:** *Biocenter, Division of Neurobiochemistry, Innsbruck Medical University, 6020 Innsbruck, Austria; †Division of Physiology, Innsbruck Medical University, 6020 Innsbruck, Austria; ‡Biocenter, Division of Clinical Biochemistry, Innsbruck Medical University, 6020 Innsbruck, Austria; §Friedrich Miescher Institute for Biomedical Research, 4058 Basel, Switzerland; ∥Biocenter, Division of Medical Biochemistry, Innsbruck Medical University, 6020 Innsbruck, Austria

**Keywords:** heat-shock protein, hypoxia, interaction, neuron, Nogo, oxidative stress, BiP, immunoglobulin heavy-chain-binding protein, CHO, Chinese-hamster ovary, CNS, central nervous system, ER, endoplasmic reticulum, EBFP2, enhanced blue fluorescent protein 2, Hsp, heat-shock protein, PFA, paraformaldehyde, PLA, proximity ligation assay, Prdx2, peroxiredoxin 2, RHD, reticulon homology domain, ROS, reactive oxygen species, RTN, reticulon, TCA, trichloroacetic acid

## Abstract

Nogo-A is the largest isoform of the Nogo/RTN4 (reticulon 4) proteins and has been characterized as a major myelin-associated inhibitor of regenerative nerve growth in the adult CNS (central nervous system). Apart from the myelin sheath, Nogo-A is expressed at high levels in principal neurons of the CNS. The specificity of Nogo-A resides in its central domain, NiG. We identified Apg-1, a member of the stress-induced Hsp110 (heat-shock protein of 110 kDa) family, as a novel interactor of NiG/Nogo-A. The interaction is selective because Apg-1 interacts with Nogo-A/RTN4-A, but not with RTN1-A, the closest paralogue of Nogo-A. Conversely, Nogo-A binds to Apg-1, but not to Apg-2 or Hsp105, two other members of the Hsp110 family. We characterized the Nogo-A–Apg-1 interaction by affinity precipitation, co-immunoprecipitation and proximity ligation assay, using primary hippocampal neurons derived from *Nogo*-deficient mice. Under conditions of hypoxic and oxidative stress we found that Nogo-A and Apg-1 were tightly co-regulated in hippocampal neurons. Although both proteins were up-regulated under hypoxic conditions, their expression levels were reduced upon the addition of hydrogen peroxide. Taken together, we suggest that Nogo-A is closely involved in the neuronal response to hypoxic and oxidative stress, an observation that may be of relevance not only in stroke-induced ischaemia, but also in neuroblastoma formation.

## Abstract 2

The nerve growth inhibitor Nogo-A selectively binds to the heat-shock protein Apg-1 and the expression levels of these two interactors are co-regulated under different forms of stress in neurons.

## INTRODUCTION

Nogo-A is possibly the best characterized of a variety of neurite outgrowth inhibitors present in CNS (central nervous system) myelin. Neutralizing its activity with function-blocking antibodies results in improved axon regrowth and functional recovery in experimental CNS lesion models of adult rodents and primates [1,2]. A multicentre clinical Phase I study of anti-Nogo-A treatment has recently been successfully completed [3]. Nogo-A is a member of the membrane-embedded RTN (reticulon) protein family and is encoded by the *Nogo/Rtn4* gene that gives rise to three major isoforms, Nogo-A, Nogo-B and Nogo-C. Nogo-A is the largest isoform owing to its unique NiG domain and is predominantly expressed in the CNS [4]. Specifically, Nogo-A is abundant in the adaxonal layers of the myelin sheath produced by oligodendrocytes [5], but is also highly expressed in principal neurons of the brain and spinal cord [5–7]. Although it is the myelin-associated Nogo-A that is believed to actively suppress the regenerative growth of injured axons and to inhibit sprouting of undamaged axons, much less is known about the functionality of neuronal Nogo-A. A series of gain- and loss-of-function studies have shown that neuronal Nogo-A appears to suppress the neuritic and synaptic plasticity in the hippocampus and the cerebellum [8–10]. Although the read-out thus seems to be inhibitory for both myelin-associated and neuronally expressed Nogo-A, it remains unclear whether or not the underlying mechanisms are the same. Interpretation of data is often complicated by the fact that Nogo-A has at least two subcellular sites of action. As is typical for a RTN protein, the majority of Nogo-A is located within the ER (endoplasmic reticulum), but a small proportion is also associated with the plasma membrane both in oligodendrocytes [11] and neurons [12]. Although there is a large body of literature describing intercellular communication by plasma-membrane-associated Nogo-A, much less is known about intracellular ER-associated Nogo-A. A central theme for intracellular Nogo-A, in fact the primordial theme for all RTNs, might be to contribute to the morphogenesis of the tubular ER [13,14]. It is interesting to note that in both scenarios (intercellular communication and intracellular ER morphogenesis) the most relevant part of Nogo-A seems to be the rather short (172 residues in mouse) RHD (RTN homology domain) at the C-terminus. A number of interacting proteins have been identified for this domain, including NgR1 [15], PirB [16], BACE1 [17], Caspr [18], DP1 [14] and other RTN proteins [12].

In contrast, surprisingly little is known about the large (806 residues in mouse) central domain, NiG, which is characteristic of the Nogo-A isoform. In order to further investigate neuronal Nogo-A, we carried out a screen for interaction partners using the NiG domain as bait. We identified Apg-1, a member of the stress-induced Hsp110 (heat-shock protein of 110 kDa) family that is expressed in the brain [19], as a novel interactor of Nogo-A. In the present study we describe this interaction and outline its functional implications.

## MATERIALS AND METHODS

### Animals

*Nogo*-deficient mice lacked the isoforms Nogo-A and -B owing to targeting of the first exon containing the start codon. This mouse line has been described elsewhere [20]. BALB/c and C57Bl/6 mice were used for hippocampal neuron cultures and biochemical experiments respectively. All experimental protocols were approved by the Austrian Animal Experimentation Ethics Board in compliance with the European Convention for the Protection of Vertebrate Animals Used for Experimental and other Scientific Purposes (ETS number 123).

### Cell culture

Adherent CHO (Chinese-hamster ovary)-K1 cells were cultured as described previously [21]. CHO-K1 cells adapted to growth in suspension were cultured in CD CHO medium (Invitrogen; 10743-029) supplemented with L-glutamine (4 mM; PAA, M11-004) and 100 units/ml penicillin/100 μg/ml streptomycin (PAA, P11-010). Cells were either grown in T-75 flasks for passaging or in spinner flasks for protein production. Seeding density was 1–2×10^6^ cells/ml and cells were passaged twice a week. Spinner flasks of 1 litre and 500 ml were used with a stirring speed of 35 rev./min in an incubator at 37°C at 8.5% CO_2_.

### Plasmids

The bait construct NiG-Strep_pAPtag5 was generated by placing the NiG domain (residues 174–979 of rat Nogo-A) into the pAPtag5 vector at the BglII and XhoI sites thereby deleting the AP-tag. On the C-terminus of NiG a triple *Strep*-tag was added by insertion of a DNA oligomer encoding the amino acid sequence (including vector-derived linker sequence) EEALSLEGPGAAGASGGGWSHPQFEKGSGGGWSHPQFEKGLWSHPQFEK-Stop at the XhoI and ApaI sites (individual *Strep*-tags underlined). NiR_pGEX-6P was described previously [11]. Nogo-B_pcDNA3 and Nogo-A_pcDNA3 were described previously [11,21]. A mouse Apg-1 expression clone was purchased from Open Biosystems (clone 4190161). pEBFP2-Nuc plasmid was purchased from Addgene (#14839). For the EBFP2 (enhanced blue fluorescent protein 2)–Apg-1 fusion protein, the Apg-1 open reading frame was cloned into the pcDNA3 vector via EcoRV and NotI, EBFP2 cDNA was released from the Addgene construct by AfeI and BglII and placed into the Apg-1_pcDNA3 construct at the EcoRV site. Nogo-A–EGFP was constructed by inserting the full-length rat Nogo-A sequence into the pEGFP-N3 vector at the XhoI and BamHI sites. Nogo-B–EGFP was generated by inserting the full-length rat Nogo-B cDNA into the pEGFP-N1 vector at the XhoI and HindIII sites.

### Pull-down assays

CHO-K1 cells grown in suspension were transiently transfected with the NiG bait plasmid using Amaxa™ Nucleofector™ technology (Lonza; program U-023). The cells were grown for 4 days and harvested by centrifugation. The cells were lysed in buffer A (50 mM Hepes, pH 7.6, 150 mM NaCl, 10% glycerol, 1% Triton X-100, 1 mM PMSF, 5 μg/ml leupeptin and 10 μg/ml aprotinin). The cleared lysate was applied to a gravity-flow column containing 50 μl of Strep-Tactin®Sepharose (IBA 2-1201-010), followed by three washing steps using buffer A. Pull-down assays were performed as described previously [22].

Recombinant NiR was prepared as described previously [11]. Pull-down assays with NiR were performed in the same way as with NiG, except for elution, which was done with 250 mM imidazole.

### MS analyses

In the gel-based approach, protein digests (using trypsin) were analysed using an UltiMate 3000 nano-HPLC system (Dionex) coupled to an LTQ Orbitrap XL mass spectrometer (ThermoScientific) equipped with a nanospray ionization source. A homemade fritless fused silica microcapillary column (75 μm internal diameter×280 μm outside diameter) packed with 10 cm of 3 μm reverse-phase C_18_ material (Reprosil) was used. The gradient (solvent A: 0.1% formic acid; solvent B: 0.1% formic acid in 85% acetonitrile) started at 4% solvent B. The concentration of solvent B was increased linearly from 4% to 50% during 68 min and from 50% to 100% during 10 min. A flow rate of 250 nl/min was applied. MS instrument settings were as follows: mass spectra were acquired in positive ion mode applying a data-dependent automatic switch between survey scan and MS/MS acquisition. Survey MS scans (from *m*/*z* 300 to 1800) were acquired in the Orbitrap with a resolution of *R*=60000; the target value was 200000; maximum ionization time was set to 20 ms. Up to three of the most intense ions per scan were fragmented in the linear trap (LTQ) using collision-induced dissociation at a target value of 20000; the maximum ionization time was set to 55 ms. Dynamic exclusion settings were: repeat count 1; repeat duration 40 s; exclusion duration 90 s. Charge state screening was enabled; unassigned and singly charged ions were excluded from MS/MS.

Protein identification was performed via Sequest, Proteome Discoverer (Version 1.2, ThermoScientific) and the NCBInr database (*Mus musculus*) accepting variable modifications carbamidomethyl (C) and oxidation (M). Specific cleavage sites for trypsin (KR) were selected with two missed cleavage sites allowed. Peptide tolerance was ±10 p.p.m. and MS/MS tolerance was ±0.8 Da. The criteria for positive identification of peptides were Xcorr>2.3 for doubly charged ions, Xcorr>2.8 for triply charged ions, Xcorr>3.3 for four-fold and higher charged ions and a false discovery rate of 0.5.

In the TCA (trichloroacetic acid)-based approach, TCA-precipitated and acetone-washed protein pellets were reduced with TCEP [tris(2-carboxyethyl)phosphine], alkylated with iodoacetamide and digested with trypsin. Peptides were injected on to a reversed-phase column for LC-MS analysis in the information-dependent acquisition mode. Electrospray ionization LC-MS/MS was performed using a Magic C_18_ HPLC column (75 μm×10 cm; Swiss BioAnalytics) with a 1200 Nano-HPLC system (Agilent Technologies) connected to a LTQ Orbitrap Velos (Thermo Scientific). The peptides were loaded on to a peptide captrap (Michrom BioResources) at a flow rate of 10 μl/min for 5 min. They were eluted at a flow rate of 400 nl/min with a linear gradient of 2–36% acetonitrile in 0.1% formic acid/H_2_O in 90 min. Information-dependent acquisition analyses were done according to the manufacturer's recommendations, i.e. 1 survey scan at 60 K resolution in the Orbitrap cell was followed by up to 20 product ion scans in the linear ion trap, and precursors were excluded for 60 s after their second occurrence. Individual MS/MS spectra containing sequence information were compared with the program Mascot against the protein sequence database Swiss-Prot 2010_09 [23]. Carboxyamidomethylation of cysteine (+57.0245) was set as a fixed modification and oxidation of methionine (+15.9949 Da), deamidation of asparagine and glutamine (+0.984016 Da) and Pyro-glu formation on N-terminal glutamine (−17.02655 Da) were set as variable. Parent tolerance was 10 p.p.m. and fragment tolerance was 0.6 Da. The enzyme specificity was set to trypsin allowing one missed cleavage site. The results were further analysed with Scaffold (Proteome Software).

### Co-immunoprecipitations

Apg-1 was co-transfected with Nogo-A or Nogo-B in adherent CHO-K1 cells using Lipofectamine™ 2000 (Invitrogen; 11668). At 2 days after transfection cells were lysed with buffer A (see above) and used for immunoprecipitations. Rabbit anti-Apg-1 antibody (6 μg; Santa Cruz Biotechnology; sc-6242), 6 μg of rabbit anti-Nogo-A/B antibody (Bianca, [11]) and 6 μg of rabbit IgG (Sigma; I5006) were mixed with 200 μg of total protein lysate. Immunoprecipitation reactions were analysed via SDS/PAGE (7.5% gels) and Western blotting on to a PVDF membrane (GE Healthcare; RPN303F). For protein detection, mouse antibodies were used {11c7 for Nogo-A [11], 1:1000 dilution; mouse anti-Apg-1 (Santa Cruz Biotechnology; sc-137027), 1:200 dilution}. Blocking agent was 5% (w/v) non-fat dried skimmed milk powder in TBS-T (TBS-Tween 20; 10 mM Tris, 150 mM NaCl and 0.2% Tween 20, pH 8.0). Horseradish peroxidase-labelled secondary antibodies (Thermo Scientific, 1:10000 dilution), ECL detection kit (GE Healthcare) and Typhoon Scanner (GE Healthcare) were used for detection.

The same protocol was applied using endogenous material. Brain lysate from a 4-week-old mouse was prepared as described previously [22] and used for immunoprecipitation reactions. Total protein (1 mg) was used to precipitate the proteins of interest. The amount and type of antibodies were the same as indicated above. In addition, we used rabbit anti-RTN1-A antibody [24], 1:10000 dilution), mouse anti-Apg-2 (Santa Cruz Biotechnology; sc-365366, 1:200 dilution) and mouse anti-Hsp105 (Santa Cruz Biotechnology; sc-74550, 1:200 dilution) antibodies for Western blotting.

### Proximity ligation assay

Adherent CHO-K1 cells were transfected with Nogo-A–EGFP or Nogo-B–EGFP and Apg-1–EBFP2 using jetPEI® (Polyplus-Transfection; 101-01N). After 2 days the cells were fixed and the PLA (proximity ligation assay) was carried out according to the manufacturer's instructions (Olink Biosciences). Briefly, fixed cells were incubated in blocking solution (3% natural goat serum, 1% BSA and 0.1% Triton X-100 in PBS) for 1 h before the following primary antibodies were added overnight at 4°C: rabbit Bianca antiserum (1:10000 dilution) and mouse anti-Apg-1 (1:100 dilution). After washing with TBS-T2 (TBS with 0.1% Triton X-100), the PLA probes anti-rabbit MINUS (Olink; 92005) and anti-mouse PLUS (Olink; 92001) were added at 1:5 in blocking solution for 1 h at room temperature (22°C). Ligation was carried out for 30 min at 37°C and amplification was done for 100 min at 37°C using the Duolink In Situ Detection Reagent Orange (Olink; 92007). Following washing steps with 1× and 0.01× Wash Buffer B (1×: 200 mM Tris/HCl, pH 7.5, and 100 mM NaCl), samples were dried and embedded in Fluorescent Mounting Medium (Dako; S3023). For the PLA with hippocampal neurons, chicken NF-H antibody was added (Neuromics; CH22104; 1:2000 dilution), followed by secondary anti-chicken antibody conjugated to CF488A dye (Sigma–Aldrich; 1:1000 dilution).

### Hippocampal neuron culture

Low-density cultures of hippocampal neurons were prepared from 17-day-old embryonic BALB/c mice as described previously [25]. Briefly, dissected hippocampi were dissociated by trypsin treatment and trituration. Neurons were plated on 18-mm-diameter poly-L-lysine-coated glass coverslips in 60-mm-diameter culture dishes at a density of 3500 cells/cm^2^. After plating, cells were allowed to attach for 2–3 h in MEM (minimal essential medium; Invitrogen; 41090-028) supplemented with 10% horse serum before transferring the coverslips neuron-side-down into a 60-mm-diameter culture dish with a glial feeder layer. Neurons and the glial feeder layer were cultured in serum-free Neurobasal medium (Invitrogen; 21103-049) supplemented with GlutaMAX™ (Invitrogen; 35050-061) and B27 supplement (Invitrogen; 17504044). Ara-C (5 μM) was added 3 days after plating to stop proliferation of non-neuronal cells.

### Chemical ischaemia

Chemical ischaemia was performed as described previously [26]. Briefly, neurons on a glass coverslip at the age of 4 days *in vitro* were transferred into ACSF (artificial cerebrospinal fluid) containing 137 mM NaCl, 5.4 mM KCl, 0.3 mM Na_2_HPO_4_, 0.22 mM KH_2_PO_4_, 33 mM sucrose, 10 mM Hepes and 2 mM CaCl_2_ supplemented with or without (for control) 10 mM sodium cyanide and 2 mM 2-deoxyglucose. Incubation was for 15 min in the tissue incubator. Neurons were washed twice with PBS and put back on to the glial feeder layer for 8 h before fixation.

### Oxidative stress

Neurons on a glass coverslip at the age of 4 days *in vitro* were transferred into glial-feeder-layer-conditioned medium and incubated with or without (for control) 70 μM H_2_O_2_ (Sigma–Aldrich) for 15 h before fixation.

### Immunocytochemistry

Cells were fixed with 4% PFA (paraformaldehyde)/5% sucrose for 20 min at room temperature, washed with PBS, incubated in blocking solution for 1 h before the following primary antibodies were added overnight at 4°C: rabbit anti-Nogo-A (Invitrogen; 366600; 1:2000 dilution), mouse anti-Nogo-A 11C7 ([11]; 1:1000 dilution), mouse anti-Apg-1 (1:200 dilution), rabbit anti-Apg-1 (1:1000 dilution) and rabbit anti-BiP (immunoglobulin heavy-chain-binding protein; Stressgen; SPA-826; 1:1000 dilution). After washing with TBS-T2, secondary antibodies were added in blocking solution at a 1:1000 dilution and incubated at room temperature for 2 h. Secondary antibodies were from Invitrogen/Molecular Probes (anti-mouse and anti-rabbit; conjugated to Alexa Fluor® dyes). After washing with TBS-T2 and Hoechst containing distilled water, cells were embedded in Fluorescent Mounting Medium (Dako; S3023). Images were made with an Axio Imager.M2 microscope (Zeiss) using Plan-Apochromat 63×/1.4 Oil and Plan-Neofluar 40×/1.30 oil objectives and analysed with ImageJ (NIH).

For partial permeabilization, neurons were briefly washed with PBS before being incubated for 25 min at room temperature in partial permeabilization buffer containing (according to [27]): 10 mM Pipes, pH 6.8, 300 mM sucrose, 100 mM KCl, 2.5 mM MgCl_2_, 1.0 mM EDTA and 12.5 μg/ml digitonin plus primary antibodies as indicated above. After washing with PBS, neurons were fixed with 4% PFA/5% sucrose for 20 min at room temperature, washed with PBS, incubated in blocking solution without Triton X-100 for 1 h before secondary antibodies, as described above, were added in blocking solution without Triton X-100 for 2 h at room temperature. After washing with PBS and Hoechst containing distilled water, cells were embedded and analysed as described above. Settings for image acquisition and image analysis were kept constant for each protein when comparing complete and partial permeabilization protocols.

### Confocal imaging

3D stacks were acquired with an SP5 confocal microscope (Leica Microsystems). We used a HCX PL APO lambda blue 63.0×, 1.40 NA (numerical aperture), oil-immersion objective. The following imaging conditions were chosen: Hoechst excitation, 405 nm; emission, 410–483 nm; CF488A/Alexa Fluor® 488 excitation, 488 nm; emission, 493–556 nm; Duolink In Situ Detection Reagent Orange/Alexa Fluor® 568 excitation, 561 nm; emission, 568–723 nm. Image stacks were acquired using the LAS AF acquisition software Version 2.1.0. according to the Nyquist criterium; pixel sizes were therefore chosen as follows: *x*,*y*=60.1 nm, *z*=125.9 nm.

### Image processing

Image deconvolution was performed using Huygens Professional Version 3.4 (Scientific Volume Imaging, Hilversum, The Netherlands) in order to improve spatial resolution using the classical maximum likelihood estimation algorithm and a theoretical point spread function.

Semi-quantitative immunocytochemistry was performed with CellProfiler. Image recording parameters were kept constant throughout all experiments. Nogo-A immunoreactive areas were defined as primary objects above a manually set intensity threshold. The sum of all pixel intensities per image was measured within the primary objects in both the Nogo-A and Apg-1 channel. By dividing the summed intensities by the number of individual neurons, we obtained the average pixel intensity per neuron per image. This analysis was chosen since higher expression levels not only increase the total pixel intensity per neuron, but also markedly increase the total immunoreactive area per neuron. Elevated expression levels lead to a more intense staining of thin neuritic processes, and these structures, which would otherwise lie below the threshold, contribute to a neuron's primary object area. Only non-apoptotic neurons characterized by an intact and non-condensed nucleus (Hoechst staining) were included in the analyses irrespective of Nogo-A immunoreactivity.

### Data analysis and statistics

Data were organized and analysed using Microsoft Excel and SPSS (SPSS Inc) statistical software respectively. Statistical significance was determined by a one-sample *t* test (difference from 1) with Holm correction for multiplicity as indicated. Correlation analysis was performed using the Pearson product moment correlation coefficient as indicated.

## RESULTS

### Nogo-A interacts with Apg-1 through NiG

**Figure 1:**
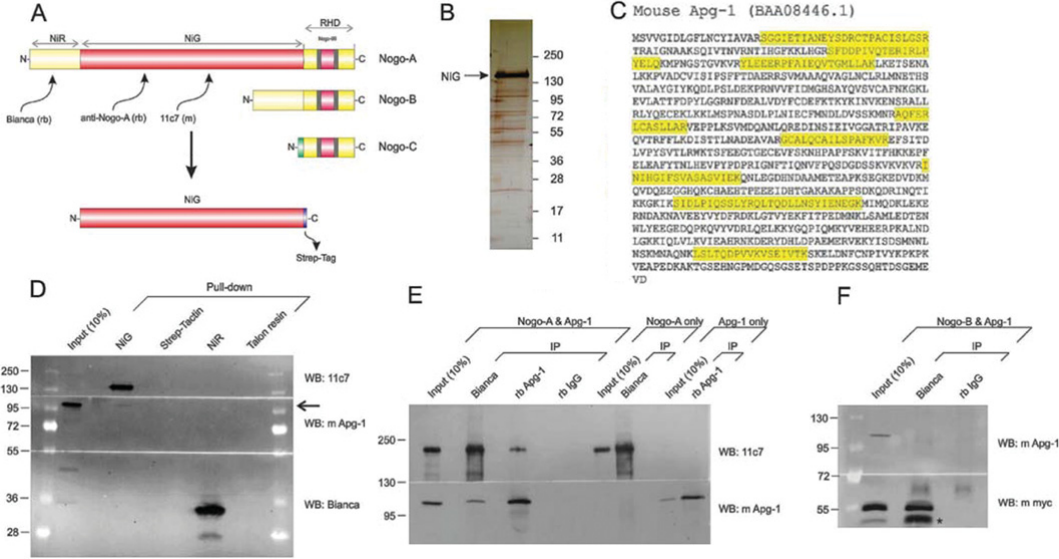
Apg-1 binds to the NiG domain of Nogo-A (**A**) Schematic diagram of the major Nogo isoforms including the domain specifications. Grey areas denote membrane embedded sequence stretches. Epitopes of antibodies used in the present study are indicated. (**B**) Silver-stained gel of recombinant NiG domain which was produced in a suspension culture of CHO-K1 cells, purified via its *Strep*-tag, and used as a bait to screen for binding proteins. (**C**) Apg-1 was identified by MS as an interactor of NiG. The peptides identified are indicated in the amino acid sequence of mouse Apg-1. (**D**) Western blot demonstrating that Apg-1 is pulled down by recombinant NiG but not NiR (arrow). Approximately 1.0 μg of recombinant NiG and NiR were used. A portion (10%) of the brain lysate that was used for pull down serves as an immunoblotting control. Respective binding matrices serve as a pull-down control. (**E**) Co-immunoprecipitation experiments with overexpressed Apg-1 and Nogo-A in CHO-K1 cells. (**F**) Co-immunoprecipitation experiments with overexpressed Apg-1 and Nogo-B in CHO-K1 cells. A prominent caspase cleavage product of Nogo-B is marked (star) [21]. A portion (10%) of the lysate of double-transfected cells that was used for co-immunoprecipitations serves as an immunoblotting control. IP, immunoprecipitation; rb, rabbit; WB, Western blot.

With its unique central NiG domain, Nogo-A is the largest of the Nogo proteins (Figure 1A). In order to identify Nogo-A-specific interactors, we produced recombinant NiG and used it as bait in an affinity chromatography approach followed by MS analysis of eluted proteins. Specifically, we produced a *Strep*-tagged NiG domain in a suspension culture of CHO-K1 cells in order to preserve native folding and post-translational modifications of the bait protein (Figure 1B). Upon immobilization of NiG, we added brain lysate from a 4-week-old mouse. The eluate was either briefly run via SDS/PAGE followed by Coomassie Blue staining and then analysed by LC-MS/MS, or it was TCA-precipitated and then analysed by LC-MS/MS. This approach produced a small number of candidate interactors of which Apg-1 was investigated further. Apg-1 was identified in three of four gel-based runs and in two of two TCA-based runs (Supplementary Table S1 at http://www.biochemj.org/bj/455/bj4550217add.htm). It was never detected in the control, which was either recombinant NiG without brain lysate, or brain lysate with mock-purification of untransfected CHO-K1 cells. We identified a total of eight peptides of Apg-1, representing a sequence coverage of 18.5% (Figure 1C).

We confirmed the pull-down of Apg-1 by Western blotting. As illustrated in Figure 1(D), Apg-1 is co-precipitated with NiG, but not with NiR, the N-terminal domain of Nogo-A/B (Figure 1A). In order to verify the interaction of Apg-1 with full-length Nogo-A, we performed co-immunoprecipitation experiments by overexpressing Nogo-A and Apg-1 in CHO-K1 cells which lack both endogenous proteins (Figure 1E). Apg-1 was readily co-immunoprecipitated by Bianca, a rabbit antibody directed towards NiR [11], but not by a control rabbit IgG. When overexpressing Nogo-B, however, Bianca did not co-immunoprecipitate Apg-1 (Figure 1F). Conversely, Nogo-A was co-immunoprecipitated by a rabbit anti-Apg-1 antibody, with the control rabbit IgG being negative. Overexpression of either protein alone did not result in any immunoreactive band of the binding partner. Overall expression levels of Nogo-A/B and Apg-1 were documented by blotting a proportion of the immunoprecipitation inputs.

Taken together, these data demonstrate that, within a heterologous expression system, Apg-1 binds to full-length Nogo-A through the NiG domain.

### Nogo-A binds selectively to Apg-1 under endogenous conditions

**Figure 2:**
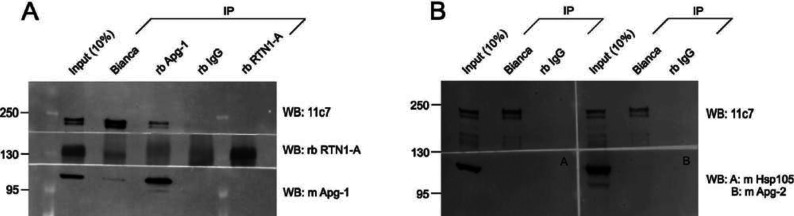
Nogo-A interacts selectively with Apg-1 under endogenous conditions (**A**) Co-immunoprecipitation experiments with endogenously expressed RTN4-A/Nogo-A, RTN1-A and Apg-1 using mouse brain lysate. (**B**) Co-immunoprecipitation experiments with endogenously expressed Nogo-A, Hsp105 (A), and Apg-2 (B) using mouse brain lysate. A portion (10%) of the brain lysate that was used for co-immunoprecipitations serves as an immunoblotting control. IP, immunoprecipitation; rb, rabbit; WB, Western blot.

We next addressed whether or not Nogo-A and Apg-1 interact under endogenous conditions in native brain tissue. Both Nogo-A [5–7] and Apg-1 [19] are abundantly expressed in the adult rodent brain. We also addressed the question of selectivity. Hsps are often found in affinity chromatography experiments since they bind to unfolded proteins, in particular to bait proteins that may partially denature during the course of overexpression. We wanted to test the selectivity of the Nogo-A–Apg-1 interaction by investigating the interactors’ affinity for closely related paralogues under native conditions. We found that Apg-1 was co-immunoprecipitated with Nogo-A/RTN4-A, but not with RTN1-A, from adult mouse brain (Figure 2A). RTN1 is the closest paralogue of Nogo/RTN4 and is also highly expressed in CNS neurons [28]. Conversely, Nogo-A was robustly co-immunoprecipitated with Apg-1 from brain tissue, but co-immunoprecipitations with a rabbit control IgG proved negative, indicating the specificity of the co-immunoprecipitations. We also tested whether Nogo-A binds to Apg-2 and Hsp105, two other members of the Hsp110 family expressed in the brain [29], but we did not find any association between Nogo-A and either of these Hsps in brain tissue (Figure 2B).

In summary, these co-immunoprecipitation experiments suggest that Nogo-A and Apg-1 interact in a specific and selective way within the native brain.

### Probing the Nogo-A–Apg-1 interaction *in vivo*

In order to exclude post-lysis co-immunoprecipitations of low physiological relevance, we tested the Nogo-A–Apg-1 interaction *in vivo*. We opted for primary hippocampal neurons because they were shown to express both Nogo-A [10] and Apg-1 [30] at a significant level. To start with, we investigated the subcellular distribution of the two proteins. As expected, Nogo-A displayed a reticular staining pattern, whereas Apg-1 showed a mainly cytoplasmic localization (Supplementary Figure S1 at http://www.biochemj.org/bj/455/bj4550217add.htm). We did not observe any redistribution of Apg-1 in the absence of Nogo-A, as illustrated by a confocal analysis of Apg-1 in hippocampal neurons derived from *Nogo*-knockout mice (Supplementary Figure S1).

**Figure 3:**
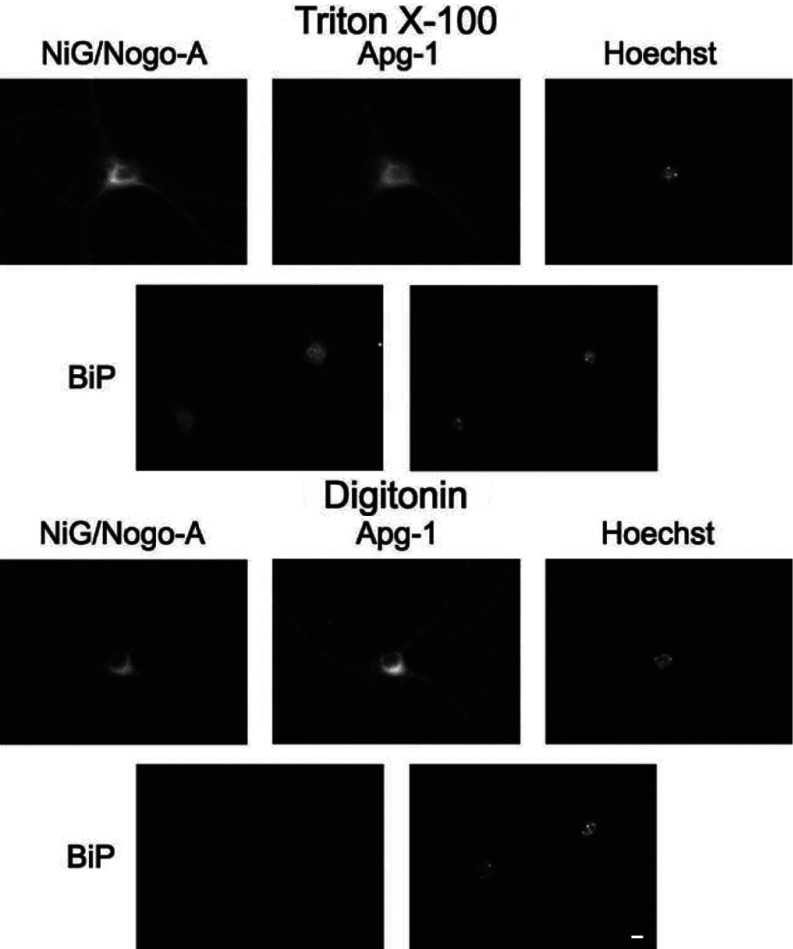
Topology of the NiG domain in primary hippocampal neurons Primary hippocampal neurons were cultured for 20 days before being fixed and completely permeabilized with 0.1% Triton X-100, followed by incubation with the 11C7 antibody, directed against the NiG domain, and anti-Apg-1 or anti-BiP antibody, or only plasma membrane permeabilized with 0.00125% digitonin in the presence of the respective primary antibodies and fixed thereafter. After selective permeabilization with digitonin, 11C7 is still immunoreactive, whereas the lumenal ER protein BiP is not, indicating a cytoplasmic localization of NiG, along with Apg-1. Scale bar, 10 μm.

We next addressed the question of the topology of neuronal Nogo-A. Specifically, we explored whether the NiG domain resides within the lumen of the ER or faces the cytoplasmic space. We performed live staining for NiG with partially permeabilized neurons using digitonin as the detergent. Digitonin at low concentrations permeabilizes solely the plasma membrane leaving all intracellular membranes intact [27]. As shown in Figure 3, NiG is readily detected in partially permeabilized neurons, along with Apg-1, whereas BiP, an ER lumenal protein, is not. These results demonstrate that both binding partners, Apg-1 and the NiG domain, reside within the cytoplasmic compartment.

**Figure 4:**
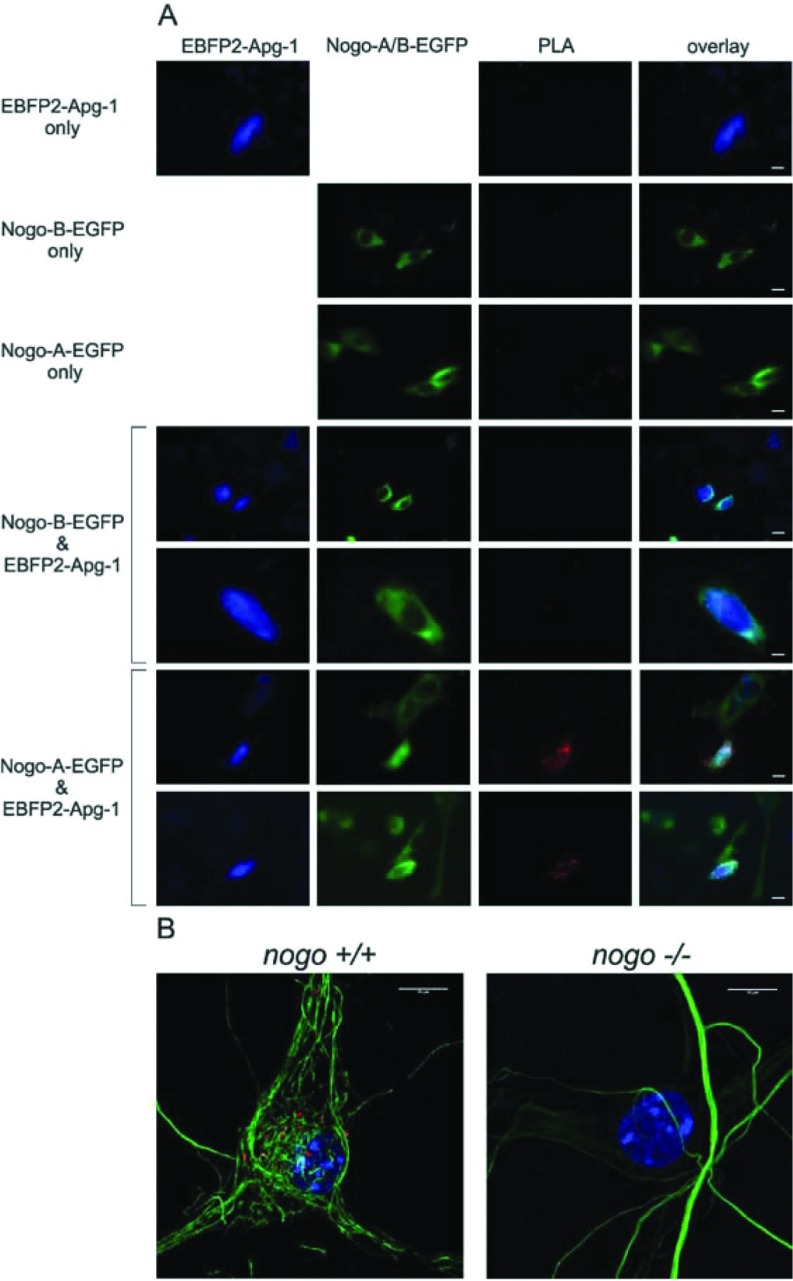
PLA with Nogo-A and Apg-1 (**A**) PLA with overexpressed Nogo-A–EGFP and EBFP2–Apg-1 in CHO-K1 cells. PLA signals (red) are specific only in double-transfected cells. Single-transfected cells as well as Nogo-B–EGFP/EBFP2–Apg1 double-transfected cells serve as a specificity control. (**B**) Confocal images of a PLA with endogenously expressed Nogo-A and Apg-1 in mouse hippocampal neurons. *Nogo^+/−^* mice were mated and hippocampal neurons from *Nogo^+/+^* and *Nogo^−/−^* embryos were cultured for 20 days before fixed and subjected to PLA. PLA signals (red) are specific only in *Nogo^+/+^* neurons. Neurons were visualized by neurofilament staining (green). Scale bars, 10 μm.

In order to check for physical interaction between Nogo-A and Apg-1 *in vivo*, we made use of the PLA. We first transfected CHO-K1 cells with Nogo-A and Apg-1, either individually or by co-transfection. We used Nogo-A–EGFP and EBFP2–Apg1 constructs, which did not exhibit any altered subcellular distribution as compared with the untagged proteins (results not shown). Upon co-transfection with Nogo-A and Apg-1, we saw bright PLA signals in double transfected cells, whereas single or untransfected cells showed only a low background of PLA signals (Figure 4A). We also included Nogo-B–EGFP as a control. In line with the negative co-immunoprecipitation experiments, we did not detect any specific PLA signal in Nogo-B/Apg-1 double- transfected cells. Finally, we tested the PLA with endogenously expressed Nogo-A and Apg-1 in hippocampal neurons. The PLA signals were lower than for the heterologous CHO-K1 expression system, but were clearly specific, as illustrated with *nogo*-knockout neurons, which lacked almost any PLA signal (Figure 4B).

Taken together, these data document the *in vivo* occurrence of the Nogo-A–Apg-1 interaction, and thus emphasize its physiological relevance.

### Nogo-A and Apg-1 are up-regulated in concert under hypoxic conditions

**Figure 5:**
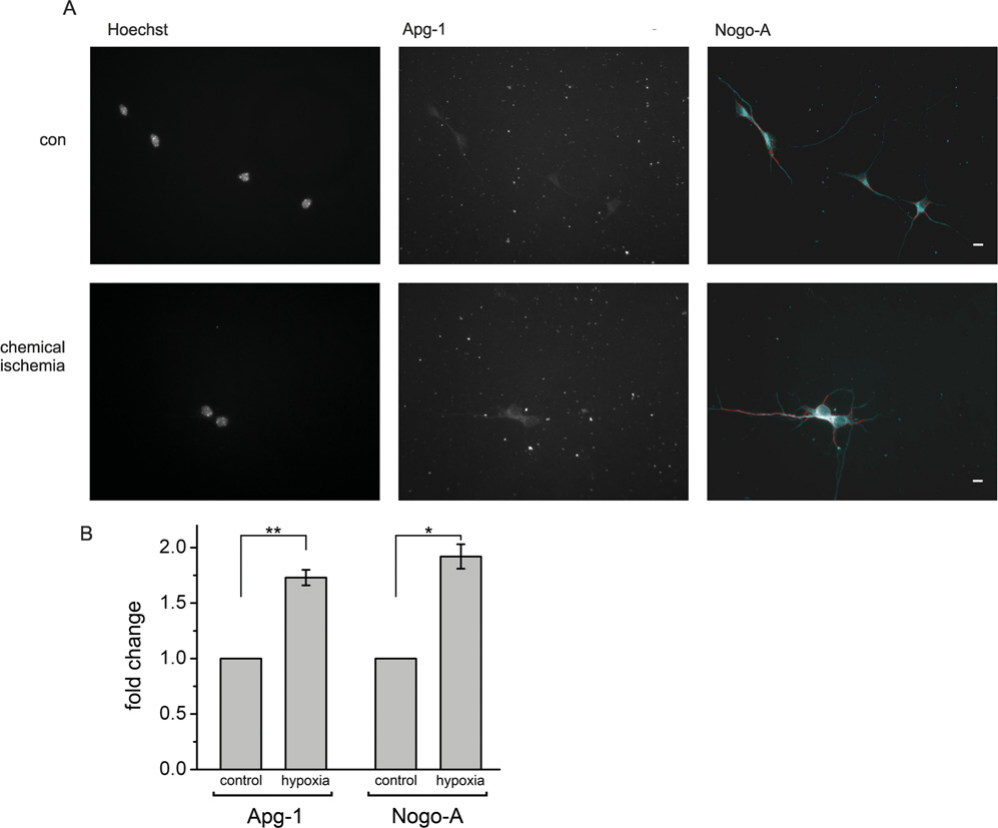
Up-regulation of Nogo-A and Apg-1 under hypoxic stress (**A**) Hippocampal neurons were cultured for 4 days *in vitro* and exposed to chemical ischaemia for 15 min, fixed after 8 h, and stained for Apg-1 and Nogo-A. Single-cell semi-quantitative immunocytochemistry was performed by defining a primary object area in the Nogo-A channel (red line). Pixel intensity was measured within this primary object area in both the Nogo-A and Apg-1 channels and related to the number of Nogo-A-positive cells with an intact nucleus to yield the pixel intensity per neuron. Scale bar, 10 μm. (**B**) Quantification of single-cell Apg-1 and Nogo-A immunofluorescence under control conditions and following chemical ischaemia. Four independent experiments with a total number of approximately 400 neurons were analysed (Apg-1 compared with control: **P=0.008, *n*=4; Nogo-A compared with control: *P=0.012, *n*=4; one-sample *t* test with Holm correction for multiplicity). con, control.

The positive PLA for Nogo-A and Apg-1 in primary hippocampal neurons prompted us to investigate further the relationship between these two proteins within this cell type. Given that Apg-1 has been described as a Hsp induced under various forms of stress [19,31], we exposed hippocampal neurons to hypoxic conditions thereby mimicking stroke-related ischaemia, which is probably the most clinically relevant stress paradigm of the brain [32]. The stress conditions were defined such that approximately 50% of the neurons died before the end of the experiment. Only surviving neurons with an intact non-condensed nucleus were evaluated in a single-cell approach, thereby excluding dead or dying neurons with degraded Nogo-A. Following a post-stress period of 8 h we observed a marked up-regulation of Nogo-A protein (Figure 5A). Likewise, Apg-1 expression was strongly increased within this time period (Figure 5A). When comparing the expression levels of the two proteins by single-cell semi-quantitative immunocytochemistry, we found a significant increase in the Nogo-A and Apg-1 staining intensity under hypoxic conditions when compared with normoxic neurons. The extent of the observed changes was similar for Nogo-A and Apg-1 (Figure 5B). We also performed a PLA under hypoxic conditions, but did not detect a change in signals when compared with the normoxic control, presumably due to the low dynamic range of this assay (results not shown).

### Nogo-A and Apg-1 are down-regulated in concert under oxidative stress

**Figure 6:**
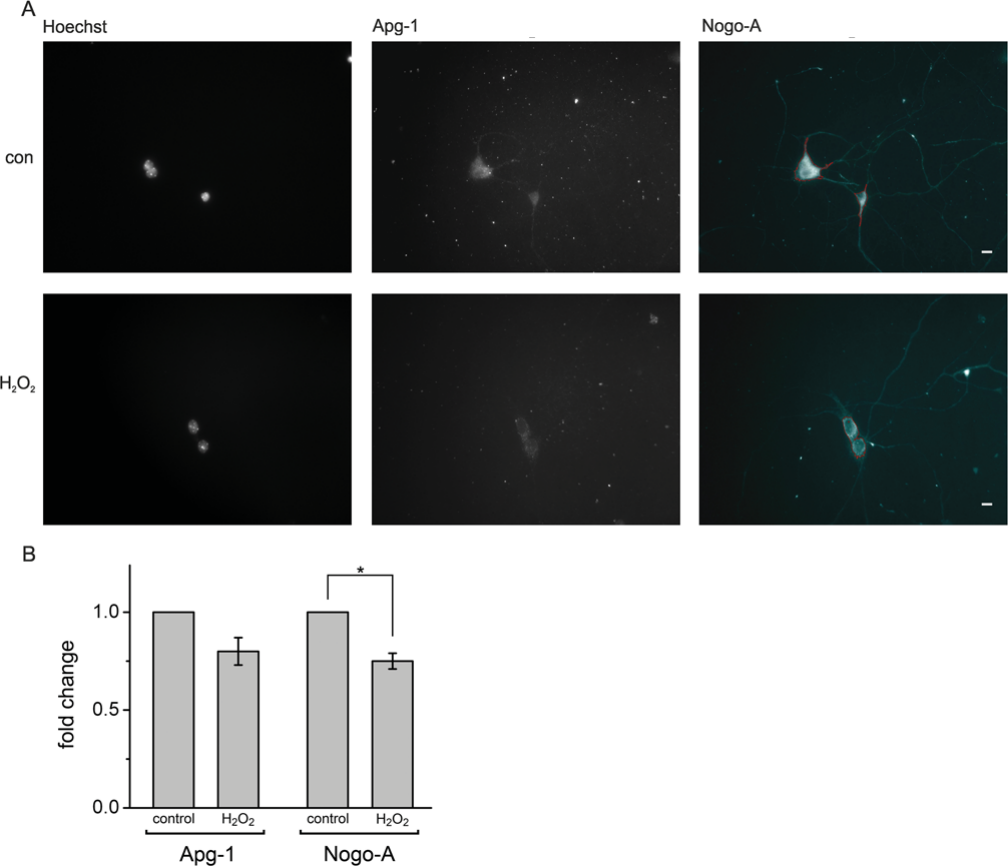
Down-regulation of Nogo-A and Apg-1 under oxidative stress (**A**) Hippocampal neurons were cultured for 4 days *in vitro* and exposed to H_2_O_2_ for 15 h, fixed and stained for Nogo-A and Apg-1. Single-cell semi-quantitative immunocytochemistry was performed by defining a primary object area in the Nogo-A channel (red line). Pixel intensity was measured within this primary object area in both the Nogo-A and Apg-1 channels and related to the number of Nogo-A-positive cells with an intact nucleus to yield the pixel intensity per neuron. Scale bar, 10 μm. (**B**) Quantification of single-cell Nogo-A and Apg-1 immunofluorescence under control conditions and following H_2_O_2_ treatment. Four independent experiments with a total number of approximately 400 neurons were analysed (Apg-1 compared with control: *P*=0.061, *n*=4; Nogo-A compared with control: *P=0.018, *n*=4; one-sample *t* test with Holm correction for multiplicity). con, control.

After examining the effect of reduced oxygen supply on Nogo-A and Apg-1 expression, we tested the neurons’ response to treatment with H_2_O_2_ as a means of inducing oxidative stress. The application of H_2_O_2_ leads to the generation of ROS (reactive oxygen species), to which neurons are very sensitive [33]. We again defined stress conditions such that approximately 50% of the neurons died by the end of the experiment and only surviving neurons with an intact non-condensed nucleus were evaluated. After 15 h of incubation with 70 μM H_2_O_2_ we observed a moderate decrease in expression levels (Figure 6A), which was significant for Nogo-A and close to significance for Apg-1 (Figure 6B). Higher concentrations of H_2_O_2_ or longer/shorter incubation periods failed to produce a more pronounced decrease in the expression levels of either protein (results not shown). Interestingly, the extent of the changes in expression levels was, once again, quite similar for Nogo-A and Apg-1 (Figure 6B).

### Nogo-A and Apg-1 expression is tightly correlated under stress

**Figure 7:**
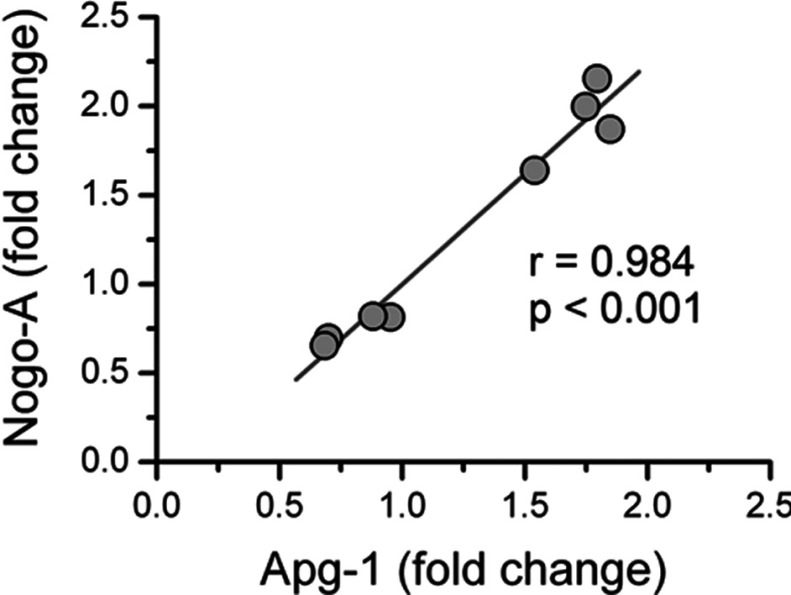
Co-ordinate expression of Nogo-A and Apg-1 under different forms of stress The hypoxic and oxidative stress experiments were pooled by plotting the fold change values of Nogo-A against the cognate fold change value of Apg-1. In the resulting scatter plot a tight correlation between the Nogo-A and Apg-1 fold change values becomes apparent with a Pearson correlation coefficient of *r*=0.984 (*P*<0.001, *n*=8) indicating a very strong direct correlation (*r*^2^=0.968) between the expression levels of Nogo-A and Apg-1 under stress.

In order to find out whether or not the changes in the expression levels of Nogo-A and Apg-1 are related to each other, we pooled the fold change data from the hypoxic and oxidative stress experiments and performed a correlation analysis. As illustrated in Figure 7 we obtained a highly significant Pearson correlation coefficient (*r*=0.984, *P*<0.001, *n*=8) indicating a very strong (*r*^2^=0.968) direct correlation between the expression levels of Nogo-A and Apg-1 under stress. This finding suggests that Nogo-A and Apg-1 are tightly linked at the expression level under hypoxic and oxidative stress, implying that these two proteins share mechanisms regulating expression and are constituents of a defined protein complex.

Taken together, these data demonstrate that hippocampal neurons are plastic with regard to expression levels of Nogo-A and Apg-1 under stress. Hypoxic and oxidative stresses elicit opposite changes in the expression levels of Nogo-A and Apg-1 within the same cell type. In addition, we have measured the highly correlated expression of Nogo-A and Apg-1 under hypoxic and oxidative stress.

## DISCUSSION

### Apg-1 binds to the NiG domain of Nogo-A

Nogo-A is best known for its role as a myelin-associated inhibitor of axonal growth in the lesioned adult CNS. In fact, attempts to delineate the inhibitory mechanisms consisting of myelin-associated ligands, neuronal receptors and co-receptors, and inhibitory signalling pathways, have largely concentrated on Nogo-A [1]. Only recently has the focus been directed towards the role of neuronal Nogo-A.

Nogo-A is markedly expressed in principal neurons of the CNS and manipulating its expression level or applying function-blocking antibodies was observed to affect neuritic and synaptic plasticity [8–10]. Although no domain analyses have been performed in either of those previous studies, the experimental design often argues for a Nogo-A specific, i.e. NiG-mediated, mechanism. In contrast with the other prominent domain of Nogo-A, Nogo-66, the role of NiG is still poorly understood for lack of explicit data.

Along with the Nogo-66 loop within the RHD, the NiG domain participates in inhibitory signalling to outgrowing axons, and integrins have been implicated in mediating this effect, although they do not appear to physically interact with NiG [34]. In a recent paper the first recognized physical interactor of NiG was identified as Prdx2 (peroxiredoxin 2), a neuronally expressed member of the peroxiredoxin family of peroxidases [35]. This interaction seems to be essential to conferring a neuroprotective role to Nogo-A by reducing the generation of ROS under H_2_O_2_-invoked oxidative stress. We now add Apg-1 to the short list of NiG-binding proteins, and it is again an interactor implicated in stress management.

Apg-1/HSPA4L/HSPH3 is one of four members of the stress-induced Hsp110/HSPH family, which is composed of Hsps of the highest molecular mass, which is approximately 110 kDa [29]. The highest expression of Apg-1 is found in the testis, with significant levels of expression also observed in the heart and brain [19]. The expression of Apg-1 is up-regulated by moderate heat shock [19], by hyperosmotic stress [36] and under ischaemic conditions [37]. Specifically, constitutive expression of Apg-1 is detected by *in situ* hybridization in the cerebral cortex and hippocampus, and transcript levels have been shown to increase markedly in these two areas in an *in vivo* ischaemia lesion model of the rat brain [37]. Two paralogues of Apg-1, Apg-2 and Hsp105, were positively tested for chaperone-like and anti-apoptotic activity [38]. Given the high degree of sequence conservation, it is very likely that Apg-1 also acts as a chaperone. Moreover, Hsp110 proteins have been reported to be nucleotide-exchange factors for Hsp70 proteins, thereby enhancing their chaperone activity [39,40]. The Hsp110 family may even have an explicit neuroprotective function; it has been demonstrated that PC12 cells overexpressing Hsp105 become more resistant to certain forms of cellular stress, including exposure to H_2_O_2_ [41].

We now put forward the hypothesis that the NiG domain acts as an anti-stress platform by recruiting a variety of proteins that help neurons cope with cellular stress, in particular with hypoxic and oxidative stress. Prdx2 may assist in this way by reducing and thus neutralizing ROS, whereas Apg-1 may prevent proteins from unfolding under stressed conditions by virtue of its presumed chaperone-like activity. In line with this concept is the fact that Nogo-A redistributes, but does not physically bind to, the ER chaperone protein disulfide isomerase, a molecular rearrangement that has been shown to convey protection against neurodegeneration [42]. Although we have not observed such a Nogo-A dependent subcellular redistribution of Apg-1 in primary hippocampal neurons, it is important to note that NiG of ER-associated Nogo-A faces the cytosol, a finding that is in line with respective data obtained with primary oligodendrocytes [11]. The cytosol, in turn, is the major subcellular compartment of Apg-1, as illustrated in the present study.

Deciphering the client specificity of Hsp110 proteins, which remains as yet unknown, will be of particular interest for future research [29]. All four members of this family are expressed in the brain and, although there is extensive overlap, each member seems to have its own enriched area of expression [30]. Hsp105, for instance, is highest in the CA3 region of the hippocampus, whereas Apg-1 and Apg-2 are enriched in the dentate gyrus. This preference of expression is reflected by a remarkable specificity of interaction with Nogo-A. Although we regularly obtained a stronger input signal on the immunoblot for Hsp105 and Apg-2 than for Apg-1, we did not detect any signal for Hsp105 or Apg-2 from a Nogo-A co-immunoprecipitation, in contrast with Apg-1, which was readily pulled-down from brain tissue by Nogo-A. This specificity is also seen in reverse; Apg-1 co-immunoprecipitated Nogo-A/RTN4-A, but not RTN1-A, the closest relative of Nogo that is also abundantly expressed in the brain [28]. The Nogo-A–Apg-1 interaction thus seems to be genuinely selective, at least in the brain, and to be specific to neurons since Apg-1 does not seem to be expressed in oligodendrocytes [37].

### Apg-1 and Nogo-A show co-ordinate expression under stress

By analysing single neurons we have found a remarkable correlation of the expression changes of Nogo-A and Apg-1 protein under different forms of stress. Tightly co-ordinated expression levels indicate functional linkage. Correlated proteins may share mechanisms of expression regulation and be constituents of a defined protein complex.

Although we have observed a marked up-regulation of Nogo-A and Apg-1 under hypoxic conditions, the down-regulation of the two proteins under oxidative stress was rather moderate. This finding is in striking contrast with the observations by Mi et al. [35] for Nogo-A in cortical neurons. Although they also noted a down-regulation of Nogo-A following treatment with H_2_O_2_, the magnitude of this effect was much greater than that we have observed. Given that conditions of stress were quite similar in these two studies, the discrepancy is presumably explained by the different techniques used for measuring Nogo-A protein levels. Although Mi et al. [35] determined Nogo-A expression levels by Western blotting, we performed single-cell semi-quantitative immunocytochemistry. In contrast with the Western blotting approach, we did not include any dead or dying neurons in our analysis, but strictly confined the evaluation to live neurons with an intact non-condensed nucleus and therefore intact, rather than degraded, Nogo-A protein.

Our results from the present study on the co-ordinate expression of Nogo-A and Apg-1 under stress are also relevant with regard to the potential cytoprotective role of these proteins in the event of stroke-induced ischaemia and neuroblastoma formation. Both Nogo-A and Apg-1 have been shown to be up-regulated in an *in vivo* ischaemia lesion model of the brain [37,43,44] and we show in the present study an *in vitro* model system which is plastic with regard to the type of stress applied and which is easily accessible to experimental manipulation. Apart from stroke-related ischaemia, there is growing appreciation of the hypoxic and oxidative stress that tumour cells are subjected to and the regulatory mechanisms they evolve in order to cope with, and even benefit from, such stress [45]. On the one hand, cells in the inner tumour mass are confronted with hypoxia for lack of appropriate oxygen supply, whereas on the other hand excessive ROS are generated because of oxidative phosphorylation failure and enhanced NADPH oxidase activity under low oxygen conditions. Tumour cells seem to be able not only to adapt to a microenvironment that would be hostile for a normal cell, but also to exploit these conditions such that the tumour cells become more aggressive and insensitive to chemotherapeutics following their adaptation than they were previously [46]. Hypoxia and ROS appear to play a key role in this scenario of adaptive microevolution and an important subject for future research will be to explore the possible relevance of Nogo-A. In fact, Nogo-A has been reported to be expressed at high levels in a number of rodent and human neuroblastoma cell lines in an apparently differentiation-dependent manner [47].

In conclusion, the present study identifies and describes the interaction of Nogo-A with Apg-1, a member of the Hsp110 family of stress-induced chaperones. We not only show an *in vivo* physical interaction between these two proteins, but also demonstrate a link at the protein expression level. Specifically, we found both proteins to be regulated in concert in primary hippocampal neurons under different forms of stress. Our data support the proposition that Nogo-A functions as an anti-stress platform through its central domain NiG, recruiting cytoprotective proteins including Apg-1 and Prdx2. This new data also extends the current view of Nogo-A as a major inhibitor of nerve growth and places a focus on its potential role in circumstances of hypoxic and oxidative stress, in particular stroke-induced ischaemia and neuroblastoma formation.

## Online data

Supplementary data
