# Supplementary material for: Nogo-A couples with Apg-1 through interaction and co-ordinate expression under hypoxic and oxidative stress
Source: Biochem J. 2013 Sep 27;455(Pt 2):217–27. doi: 10.1042/BJ20130579 (PMC3806365; doi:10.1042/BJ20130579)
Supplement: Supplementary data [file bj4550217add.pdf]

Biochem. J. (2013) **455**, 217–227 (Printed in Great Britain) doi:10.1042/BJ20130579

SUPPLEMENTARY ONLINE DATA

Nogo-A couples with Apg-1 through interaction and co-ordinate expression under hypoxic and oxidative stress

Florian KERN*, Ruslan I. STANIKA†, Bettina SARG‡, Martin OFFTERDINGER*, Daniel HESS§, Gerald J. OBERMAIR†, Herbert LINDNER‡, Christine E. BANDTLOW*, Ludger HENGST|| and Rüdiger SCHWEIGREITER*[1]

*Biocenter, Division of Neurobiochemistry, Innsbruck Medical University, 6020 Innsbruck, Austria, †Division of Physiology, Innsbruck Medical University, 6020 Innsbruck, Austria, ‡Biocenter, Division of Clinical Biochemistry, Innsbruck Medical University, 6020 Innsbruck, Austria, §Friedrich Miescher Institute for Biomedical Research, 4058 Basel, Switzerland, and ||Biocenter, Division of Medical Biochemistry, Innsbruck Medical University, 6020 Innsbruck, Austria

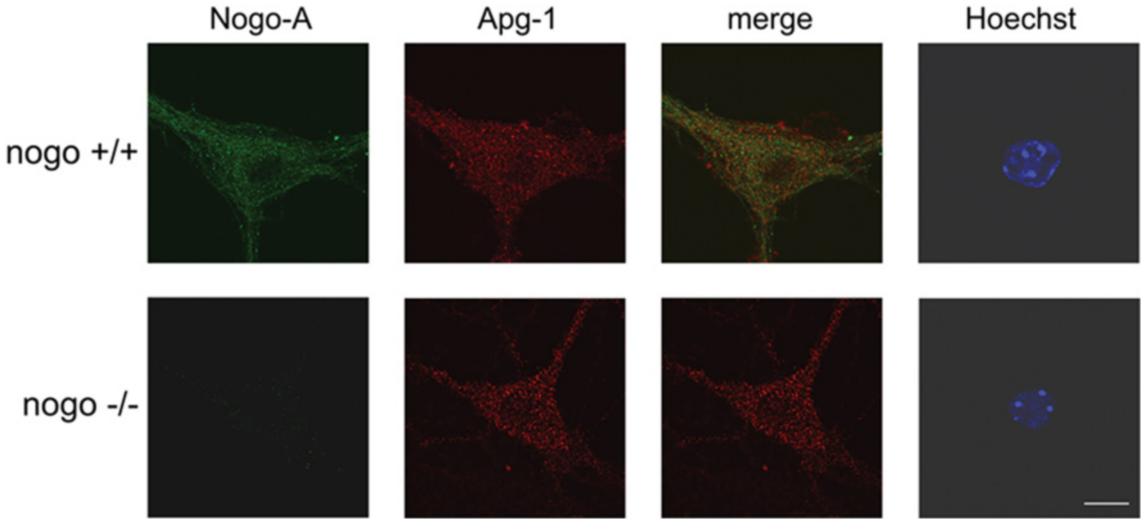

Figure S1 Subcellular localization of Nogo-A and Apg-1 in cultured hippocampal neurons

Hippocampal neurons from wild-type and *Nogo*$^{-/-}$ mice were cultured for 20 days *in vitro* before being fixed and stained for Nogo-A and Apg-1. Single-plane confocal analyses illustrate the subcellular localization of these two proteins in the wild-type and of Apg-1 in the *Nogo*-knockout. Scale bar, 10 μm.

[1] To whom correspondence should be addressed (email ruediger.schweigreiter@i-med.ac.at).

© 2013 The Author(s)
The author(s) has paid for this article to be freely available under the terms of the Creative Commons Attribution Licence (CC-BY) (http://creativecommons.org/licenses/by/3.0/) which permits unrestricted use, distribution and reproduction in any medium, provided the original work is properly cited.

Biochemical Journal www.biochemj.org

Table S1 List of NiG candidate interactors identified by two different MS screens

All proteins are listed which were identified in two independent MS screens as NiG-interactor candidates. Following elution from the NiG column, proteins were either subjected to brief SDS/PAGE, followed by in-gel digestion and LC-MS/MS (four independent experiments), or were TCA-precipitated and, after digestion, subjected to LC-MS/MS (two independent experiments). The criteria for inclusion were at least two hits with the gel-based approach without any detection in the controls, and two hits with the TCA-based approach without any detection in the controls. Two peptides per protein were the minimum for a positive protein identification. Peroxiredoxins were addressed separately because Prdx2 was described as an interactor of NiG [1]. As shown in the Table, we have also found peroxiredoxins, including Prdx2, in both MS screens, albeit with a lower frequency and/or weaker sample compared with the control ratio than other proteins. CaMKII (Ca^{2+}/calmodulin-dependent protein kinase II) had a very good frequency and sample compared with control ratio in the gel-based approach, but we could not confirm interaction with endogenous Nogo-A via co-immunoprecipitation, suggesting that CaMKII interacts with the isolated NiG domain, but not with full-length Nogo-A.

Screen	Protein name	Accession number	Frequency of hits (sample/control)	Remarks
Gel approach (four independent experiments)	CaMKIIα	EDL09793.1	4×/0×	We could not verify interaction via co-immunoprecipitation using brain lysate
	Apg-1	NP_035150	3×/0×	Interaction is characterized in the present paper
	Clathrin heavy chain 1	NP_001003908, XP_181312	3×/0×	
	F-actin capping protein α	EDL13878	2×/0×	
	Dynamin 3	EDL39302.1	2×/0×	
	Protein phosphatase 1, regulatory subunit 7	EDL39961	2×/0×	
	Prdx1	NP_035164	2×/2×	
	Prdx4	AAH19578	1×/0×	
TCA approach (two independent experiments)	Apg-1	NP_035150	2×/0×	Interaction is characterized in the present paper
	Prdx1	NP_035164	2×/2×	
	Prdx2	NP_035693	2×/1×	Interaction with NiG/Nogo-A was described in [1].
	Prdx5	P99029	1×/0×	

REFERENCE

1 Mi, Y. J., Hou, B., Liao, Q. M., Ma, Y., Luo, Q., Dai, Y. K., Ju, G. and Jin, W. L. (2012) Amino-Nogo-A antagonizes reactive oxygen species generation and protects immature primary cortical neurons from oxidative toxicity. Cell Death Differ. **19**, 1175–1186

Received 26 April 2013/30 July 2013; accepted 2 August 2013
Published as BJ Immediate Publication 2 August 2013, doi:10.1042/BJ20130579

© 2013 The Author(s)
The author(s) has paid for this article to be freely available under the terms of the Creative Commons Attribution Licence (CC-BY) (http://creativecommons.org/licenses/by/3.0/) which permits unrestricted use, distribution and reproduction in any medium, provided the original work is properly cited.
